# Perampanel in Brain Tumor-Related Epilepsy: A Systematic Review

**DOI:** 10.3390/brainsci13020326

**Published:** 2023-02-14

**Authors:** Payam Tabaee Damavandi, Francesco Pasini, Gaia Fanella, Giulia Sofia Cereda, Gabriele Mainini, Jacopo C. DiFrancesco, Eugen Trinka, Simona Lattanzi

**Affiliations:** 1 Department of Neurology, Fondazione IRCCS San Gerardo dei Tintori, School of Medicine and Surgery, Milan Center for Neuroscience, University of Milano-Bicocca, 20900 Monza, Italy; 2 Department of Neurology, Christian Doppler Klinik, Paracelsus Medical University, 5020 Salzburg, Austria; 3 Center for Cognitive Neuroscience, 5020 Salzburg, Austria; 4 Public Health, Health Services Research and HTA, University for Health Sciences, Medical Informatics and Technology, 6060 Hall in Tirol, Austria; 5 Neurological Clinic, Department of Experimental and Clinical Medicine, Marche Polytechnic University, 60020 Ancona, Italy

**Keywords:** perampanel, AMPA receptors, brain tumor, epilepsy, glutamate, glioma, seizure, glioblastoma

## Abstract

Brain tumor-related epilepsy (BTRE) is a common comorbidity in patients with brain neoplasms and it may be either the first symptom or develop after the tumor diagnosis. Increasing evidence suggests that brain tumors and BTRE share common pathophysiological mechanisms. Glutamatergic mechanisms can play a central role in promoting both primary brain tumor growth and epileptogenesis. Perampanel (PER), which acts as a selective antagonist of glutamate α-amino-3-hydroxy-5-methyl-4-isoxazolepropionic acid (AMPA) receptors, may play a role both in the reduction in tumor growth and the control of epileptiform activity. This systematic review aimed to summarize the pre-clinical and clinical evidence about the antitumor properties, antiseizure effects and tolerability of PER in BTRE. Eight pre-clinical and eight clinical studies were identified. The currently available evidence suggests that PER can be an effective and generally well-tolerated therapeutic option in patients with BTRE. In vitro studies demonstrated promising antitumor activity of PER, while no role in slowing tumor progression has been demonstrated in rat models; clinical data on the potential antitumor activity of PER are scarce. Additional studies are needed to explore further the effects of PER on tumor progression and fully characterize its potentialities in patients with BTRE.

## 1. Introduction

Brain tumor-related epilepsy (BTRE) is common in patients with brain neoplasms, being the onset symptom in 15–30% of cases, and presenting later in the course of the disease in a further 20–45% of patients [1,2]. Brain tumor-related epilepsy severely impacts the morbidity and quality of life of affected people, yet its pathogenesis remains poorly understood. Of note, BTRE is drug-resistant in up to 15 to 25% of cases in high-grade gliomas, and choosing the best antiseizure medication (ASM) might be challenging in this more vulnerable population due to drug toxicity and interactions with antineoplastic agents [3].

Brain neoplasms such as high-grade gliomas have few therapeutic options. The standard medical treatment for high-grade gliomas is concomitant chemotherapy with temozolomide and radiotherapy. Even if current therapeutic approaches can improve progression-free survival, the prognosis of patients with high-grade gliomas still remains poor with the 5-year survival rate rarely reaching 5%; innovative therapies are, hence, needed [4].

There is evidence suggesting that brain tumors and tumor-associated epilepsy can share pathophysiological mechanisms: tumor growth can promote the generation of seizures which, in turn, can drive tumor progression. Glutamatergic mechanisms including the altered expression of glutamate transporters, excessive glutamate release and glutamate receptors activation, and increased extracellular glutamate concentrations can play a key role in promoting both primary brain tumor growth and epileptogenesis [5].

In glioma cells, α-amino-3-hydroxy-5-methyl-4-isoxazolepropionic acid (AMPA) receptors for glutamate are often overexpressed. By activating oncogenic signaling cascades and cytoskeletal remodeling, glutamate stimulates autocrine and paracrine responses in glioma cells, which lead to tumor growth and invasion [6,7]. While the pathophysiology of gliomas and cancer-associated epilepsy remains unclear, it may be speculated that AMPA receptor antagonists may have both anticonvulsant activity and antitumor effects. Furthermore, several cancer cell lines, including lung carcinoma, astrocytoma, neuroblastoma, and rhabdomyosarcoma/medulloblastoma, were observed to be more sensitive to conventional cytotoxic chemotherapy when AMPA receptors were blocked [8].

This insight into the role of glutamate in brain tumor growth has pointed to promising therapeutic options to treat tumor-related seizures and exert additional antitumor effects. In a phase II trial in patients with newly diagnosed glioblastoma, talampanel, an AMPA receptor antagonist, has been associated with longer survival when combined with temozolomide and radiotherapy compared to treatment with temozolomide and radiotherapy alone [9]; these findings, however, have not been confirmed in a second phase II trial [10].

Perampanel (PER) is a non-competitive AMPA receptor antagonist with a five-fold longer half-life than talampanel and good blood-brain barrier penetration. The drug has been approved in Europe and the USA as an adjunctive treatment of focal seizures in patients aged ≥ 4 years (and as monotherapy in the USA), and as an adjunctive treatment of primary generalized tonic-clonic seizures associated with idiopathic generalized epilepsy in patients aged ≥ 12 years (and ≥7 years in Europe). Given its action as a selective antagonist of glutamate AMPA receptors and the role of altered glutamate homeostasis in the migration and invasion of brain tumor cells, PER may have a role both in the reduction in tumor growth and the control of epileptiform activity.

Here, we perform a systematic review of the pre-clinical and clinical evidence about the antitumor properties, the antiseizure effects, and the tolerability of PER in BTRE.

## 2. Materials and Methods

We performed a systematic literature search using MEDLINE (accessed by Pubmed), Embase, the Cochrane Central Register of Controlled Trials (CENTRAL), and the US National Institutes of Health Clinical Trials Registry (http://www.clinicaltrials.gov (accessed on 22 December 2022)) from inception to week one of October 2022 (update on week one of February 2023). The search strategy included “perampanel”, “brain tumor” and “brain neoplasm” as keywords in different combinations using Boolean operators; no filters were applied. Duplicates, reviews, and articles in languages other than English were excluded. The risk of bias in any included clinical trial was assessed using the RoB 2 tool [11], whilst it was not assessed individually for other study types (observational cohort studies and case series/case reports) that, instead, were considered at high risk of bias [12]. This systematic review is reported according to the recommendations of the preferred reporting items for systematic reviews and meta-analyses (PRISMA) statement [13].

## 3. Results

**Figure 1:**
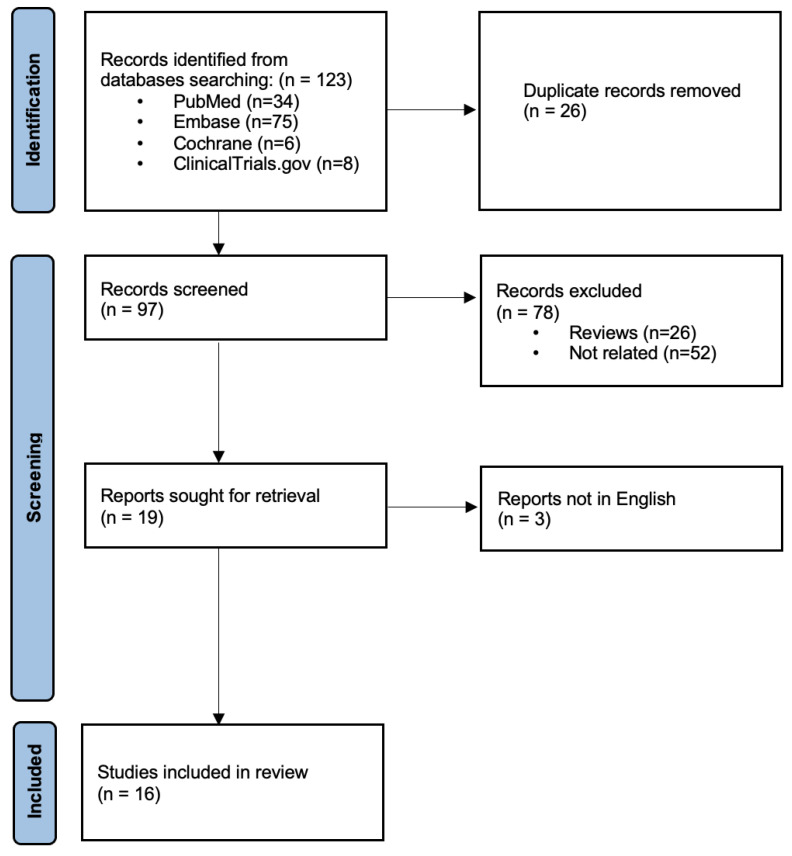
Flow diagram of study selection process.

We identified 123 records through database and trial registers searching. After the removal of duplicates, we screened 97 records. Of these, 78 were excluded as they were considered ineligible (reviews, not related to the topic) and three were excluded because the full texts were not in any of the languages reported in the inclusion criteria. We assessed 16 reports for eligibility, which were subsequently included in the review (Figure 1): eight were pre-clinical and eight were clinical studies. The clinical studies included were observational studies (four prospective, two retrospective) and two case series and were hence, considered at high risk of bias; no clinical trials were identified.

### 3.1. Pre-Clinical Studies

One of the first experimental studies available in the literature was performed by Cunningham and colleagues [14], and it showed that PER was able to block inter-ictal discharges in ex vivo human peritumoral brain slices.

Lange and colleagues [15] used patient-derived low-passage cell lines of glioblastoma and metastasis cells to study the biological and molecular effects of PER, levetiracetam, valproate, and carbamazepine on brain tumor cells. Perampanel was the only drug that showed antiproliferative effects on glioblastoma cell lines, whereas carbamazepine, valproate, and levetiracetam failed to decrease proliferation. The antiproliferative effects of PER were not explained by enhanced apoptosis, as shown by cell cycle analysis and caspase activity assay, but rather by its effect on cell metabolism, as evidenced by decreased glucose uptake in glioblastoma cells. In addition, PER decreased glutamate levels in all cell lines. To determine how PER could affect glutamate release and metabolism, a real-time PCR analysis was performed. Among the genes studied, PER showed a transcriptional effect in glioblastoma cells by increasing the expression of *BCAT1* and *GLUL*. While *BCAT1* increases intracellular glutamate levels, promoting glioma proliferation [16], *GLUL* converts glutamate to glutamine, decreasing cytosolic glutamate levels [17]. Conversely, there were no transcriptional effects on gene expression in metastasis cell cultures.

Lai and colleagues [18] examined the effects of PER on sodium currents in various cell types, including U87 glioma cells. According to this study, the peak and late components of voltage-gated Na+ currents (I_Na_) in glioma cells decreased after exposure to 1 and 3 µM PER.

Salmaggi et al. [19] evaluated the impact of PER alone or in combination with temozolomide on the growth of glioblastoma cell lines U87, U138, A172, and the grade III astrocytoma cell line SW1783. Consistent with the results of Lange [15], PER showed antitumor activity in all cell lines, and the combination of PER and temozolomide had a significant synergistic effect, with the A172 line being the most sensitive. The antitumor activity was related to a pro-apoptotic effect which occurred to varying degrees in all four glioma cell lines and was more striking at higher doses of the drug. Perampanel has been also shown to upregulate the expression of several GluR subunits in both U87 and U138 cells.

Tatsuoka et al. [20] used six malignant glioma cell lines (A-172, AM-38, T98G, U-138MG, U-251MG, and YH-13) to assess the antitumor effect of PER. The drug showed inhibitory effects on cell viability in a dose-dependent manner on all cell lines at 72 h. Five of the six cell lines exhibited significantly reduced cell viability with 1.0 µM PER treatment. At this dosage, no significant change was observed in the proportions of cells in the G0/G1, S, and G2/M phases in the T98G and U-251MG lines, which indicated that the inhibitory effect may not be due to the accumulation of cells at specific cell cycle phases. On the other hand, the protein expression level of cleaved caspase-3 was markedly greater in the PER group compared with the control, suggesting a pro-apoptotic role of the drug. Of note, in the A-172, T98G, and U-251MG cell lines, the combination of temozolomide and PER demonstrated significant additive inhibitory effects on cell viability. The same research team investigated the antitumor effects of PER, carbamazepine, valproate, and levetiracetam at therapeutic blood concentrations on six cell lines [21]. Perampanel demonstrated significant inhibition of cell proliferation in all cell lines; the same effect was observed in three cell lines treated with carbamazepine, four cell lines treated with valproate, and two cell lines treated with levetiracetam [21]. The combined antitumor effects of temozolomide were further explored using T98G and U-251MG cell lines. The results confirmed the presence of such effects in both cell lines treated with PER, as well as in T98G cells treated with levetiracetam; no such effects were observed in cells treated with carbamazepine and valproate [21]. Additionally, PER suppressed cell migration in T98G and U-251MG cells acting on the expression of different genes involved in cell migration, adhesion, and infiltration [21].

Mayer and colleagues [22] were the first to study the effects of PER on glioma C6 cells both in vitro and in vivo. In their experiment, PER reduced glucose uptake in vitro without affecting extracellular glutamate levels. Additionally, PER prevented recurrent epileptiform discharges in brain slices from animals bearing C6 glioma, and these effects were shown to be AMPA-receptor dependent. In vivo, daily therapy with PER was associated with a trend toward reduction in tumor size, although without reaching statistical significance or affecting animal survival.

Lange et al. [23] also assessed the neuroprotective potential of PER as an add-on treatment to standard radiochemotherapy in a rodent glioma model. First, they used orthotopically cultured F98 glioma cells in Fischer rats as a model of glioma progression with an epileptiform phenotype. While radiochemotherapy reduced tumor size, the addition of PER had no effect on tumor progression. However, when PER was co-administered with standard radiochemotherapy, glutamatergic network activity was maintained in healthy peritumoral tissue significantly more efficiently than standard radiochemotherapy or PER alone in rats bearing F98 glioma. Furthermore, PER showed a significant anticonvulsant effect regardless of whether it was co-administered with radiochemotherapy.

The main findings of the pre-clinical studies are summarized in Table 1.

### 3.2. Clinical Studies

#### 3.2.1. Anti-Seizure Effects of Perampanel

In all the included clinical studies, PER was given as an add-on ASM in adults with BTRE and uncontrolled seizures.

In the first study investigating the efficacy of PER in BTRE patients, Vecht and colleagues [24] reported an objective seizure response in nine out of 12 patients (75%), with a seizure frequency reduction greater than 50% in three patients and seizure freedom in six patients.

In a small case series of 12 patients, Izumoto et al. [25] reported a ≥50% responder rate, defined as a reduction in seizure frequency ≥ 50%, in all the patients included, with six patients (60%) reaching seizure freedom; two patients discontinued PER treatment.

Dunn-Pirio and colleagues [26] conducted a single-arm prospective study on PER as an add-on treatment in patients with focal-onset glioma-associated seizures. Six out of eight subjects observed a benefit from adjunctive PER in terms of seizure reduction, even though no details about the entity of seizure reduction were provided and an unspecified improvement in seizure control was reported. The authors highlighted how most patients who had a decrease in seizure activity had IDH1 mutant gliomas. However, sampling error cannot be excluded because of the high rate of IDH1 mutant tumors in the study population.

Chonan et al. [27] evaluated the efficacy of low-dose PER as the first add-on treatment in glioma patients who had uncontrolled seizures while receiving levetiracetam. Seventeen out of 18 patients became seizure-free at a median follow-up of 10.6 (range 1–21) months and a dose of 2–4 mg PER added to levetiracetam. The median time to achieve seizure freedom was 11 days (range 0–2 months). No significant difference was observed in PER efficacy according to the presence of IDH1 mutation.

In a small retrospective study, Maschio and colleagues [28] demonstrated a ≥50% responder rate of 81.8% after 12 months of treatment with PER in BTRE patients with uncontrolled seizures. A higher responder rate was observed in IDH1-mutated compared to IDH1-wild-type patients.

Subsequently, the same group of research [29] conducted a small prospective pilot study in 26 patients with uncontrolled BTRE and found a ≥50% responder rate of 95.2% at 6 months. As opposed to previous results, no significant differences in seizure control were shown in patients with/without IDH1 mutation and with/without MGMT methylation.

The multicenter, observational PERADET study [30] reported a ≥50% responder rate of 90.4% at 12 months, with 33.3% of patients being seizure-free in the per-protocol population; in the intent-to-treat analysis, the responder rate was 66.6% at 12 months, with 25% of patients being seizure-free. Patients with IDH1 mutation and patients with MGMT methylation seemed to respond better to PER treatment; the molecular analysis, however, was not available in all included patients.

In a small case series about the efficacy of PER in BTRE patients by Heugenhauser et al. [31], PER was effective to reduce seizure frequency in four of the five patients with uncontrolled BTRE; a ≥50% seizure frequency reduction was observed in two patients (40%). Interestingly, PER improved seizure control also in two patients with SMART (stroke-like migraine attacks after radiation therapy) syndrome.

The main findings about the antiseizure activity of PER in clinical studies are reported in Table 2.

#### 3.2.2. Antitumor Effects of Perampanel

Only one clinical study evaluated the potential antitumor effect of PER through MRI volume analysis of tumor lesions [25]. Izumoto and colleagues assessed the efficacy of PER on seizures and tumor progression in 12 glioma patients with uncontrolled epilepsy. All the patients had a glial tumor type, presented recurrent seizure activity at the time of initiating PER, and had used at least one ASM before the treatment with PER. All patients with malignant glioma had been treated with the Stupp regimen before PER initiation. Tumor progression was assessed within 6 months with analysis of tumor volume and peritumoral edema on FLAIR MRI. Volumetric imaging analysis showed a decreased volume of hyperintense lesions in eight patients, suggesting an inhibitory effect of PER on tumor progression and/or peritumoral brain edema. Furthermore, volume reduction showed a correlation with the plasma concentration of PER. The results of this small study might have been affected by concurrent antitumor treatment: of note, six patients received temozolomide and three patients received bevacizumab along with PER.

#### 3.2.3. Tolerability and Safety of Perampanel

Across the clinical studies, PER was generally titrated slowly by starting at the dose of 2 mg once a day for 2–4 weeks and then increasing the dose by 2 mg every 2–4 weeks to reach seizure control and/or follow the study protocol. Dunn-Pirio et al. [26] originally proposed a fast-titration approach up-titrating PER by 2 mg every week; the study, however, required a protocol amendment and a 2-week up-titration schedule was considered after side effects (fatigue and dizziness) were experienced by four patients.

In the included studies, the overall prevalence of adverse effects in BTRE patients treated with PER was quite variable, ranging from 11% [27] to 50% [24]. The most experienced adverse effects were dizziness/vertigo (18% of all patients treated with PER), fatigue (9%), aggressiveness/irritability/agitation (9%), and anxiety (5%). Other reported adverse effects were confusion, insomnia, nausea, and somnolence/drowsiness. Adverse effects of PER are generally reported to increase with increasing doses [32]. A total of eight patients [25,26,28,29,30] required a down-titration of PER because of tolerability issues: adverse effects improved following the decrease in PER dosage, and all patients continued the treatment. In the included studies, the maximum daily dose of PER reached 12 mg, reported in four patients [24,29,30]: two patients experienced no adverse effects while for the other two, no data were available. Regarding the known risk of hepatotoxicity associated with PER, only Heugenhauser et al. [31] reported measuring laboratory hepatic parameters in their cohort, showing no relevant changes.

The main findings about the tolerability of PER in clinical studies are summarized in Table 3.

## 4. Discussion

### 4.1. Pre-Clinical Evidence

**Figure 2:**
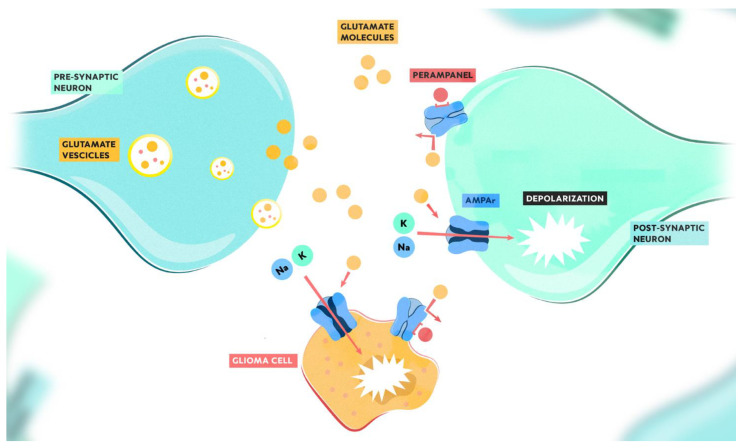
Mechanism of action of perampanel and its potential role in antitumor growth.

Pre-clinical studies suggest that epileptogenesis and tumor growth in gliomas share common pathophysiological mechanisms through glutamatergic pathways and the activation of AMPA receptors, supporting the potential beneficial effects of glutamate receptor antagonists [23] (Figure 2).

Through the upregulation of glutamate receptors—including AMPA—and post-synaptic structural genes, glioma cells establish synaptic connections. The neuronal electrochemical signaling results in the release of glutamate in the synaptic cleft. Glutamate molecules bind the AMPA receptors and depolarize glioma cells, thus promoting tumor proliferation, invasiveness, and metastatic colonization. Perampanel non-competitively binds AMPA receptors, inhibiting the sodium and potassium cytosolic influx and blocking the depolarization of both neuronal and glioma cells.

The pre-clinical studies provided preliminary evidence that PER may have anti-tumor potentialities. Several in vitro studies demonstrated that PER exhibits antitumor activity, which is increased in the presence of temozolomide in some of the malignant glioma cell lines. The mechanisms by which this activity is mediated are not clear yet. According to Lange’s study [15], PER acts on cell metabolism by decreasing glucose uptake in glioblastoma cells, without causing apoptosis. Conversely, a pro-apoptotic effect has been demonstrated in the studies by Salmaggi and Tatsuoka [19,20]. The discrepancy between these studies regarding the presence of pro-apoptotic activity can be explained by the different methods used to detect it. In the study by Salmaggi et al., PER upregulated the expression of several GluR subunits in two different glioma cell lines. The modulation of GluR subunits could decrease the permeability of glioma cells to calcium, leading to the inhibition of cell migration and the induction of apoptotic cell death [33]. Of note, in the study by Tatsuoka et al., the pro-apoptotic effect was obtained with concentrations lower than those employed by Salmaggi et al. Further research is needed to better understand how apoptosis is affected; interestingly, the role of PER on the expression of caspase-8, caspase-9, and poly(ADP-ribose) polymerase (PARP) or in the presence of caspase inhibitors has not been investigated yet [20].

Tatsuoka et al. also demonstrated that the combination of PER with tiplaxtinin further decreased cell viability in cells with high expression levels of SERPINE1 that were resistant to PER. Tiplaxtinin inhibits SERPINE1, a serine protease inhibitor that has been proposed as a factor for tumor migration and invasion in several types of cancer, including gliomas [34]. These findings overall suggest that the antitumor effect of PER may be diminished in malignant gliomas with higher expression levels of SERPINE1. Interestingly, Yagi et al. showed that the antitumor effect of PER can result not only from the inhibition of cell proliferation but also from the inhibition of cell migration: PER reduced the expression of Rac1, RhoA, and N-cadherin, which are involved in promoting cell motility, and increased the expression of E-cadherin, which has opposing effects. [21]. The mechanisms underlying the inhibitory effect on cell growth exerted by other ASMs were not examined in detail [21].

In addition, in the study by Lai and colleagues [18], PER suppressed voltage-gated Na+ currents in glioma cells. It is known that gliomas are characterized by the differential expression of voltage-gated sodium channel subtypes, which determine the expressed I_Na_ phenotype [35]. A few studies have shown that some agents acting as inhibitors of voltage-gated sodium channels can prolong the survival of patients with malignant gliomas [36,37]. Further studies are needed to determine whether the blocking effect of PER on the transient and persistent components of I_Na_ can have a synergistic and beneficial role in patients with BTRE.

While several studies have demonstrated the antitumor activity of PER in vitro, no role of the drug in slowing tumor progression has been demonstrated in rat models so far. However, in the study by Lange et al. [23] PER showed a potential neuroprotective role when co-administered with standard radiochemotherapy since it preserved the glutamatergic network activity in healthy peritumoral tissue. Additionally, in the study conducted by Mayer et al. [22] on a C6 glioma rodent model, the lack of tumor inhibitory effect of PER could be related to a too-low administered dose. Moreover, while C6 cells exhibit moderate amounts of AMPA receptors in vitro [38,39], it is not yet known whether the glutamatergic pathway plays a role in tumor progression in vivo. Given the limited available evidence, more research and in vivo experiments with rodents are needed to investigate the possible antitumoral properties of PER. Similarly, more studies are warranted to investigate how PER works as an anticonvulsant agent in the long-term in rodents and the side effects at different therapeutic doses.

### 4.2. Clinical Evidence

Since the publication of an anecdotal case report in 2015 [40] in a patient with IDH-1 wild-type glioblastoma-associated epilepsy, an increasing number of observational small studies exploring the efficacy of PER in BTRE have been published.

In all the clinical studies where the seizure baseline was calculated, PER given as add-on treatment in BTRE patients has been shown to be highly efficacious in reducing seizure frequency, with a cumulative ≥50% responder rate of 86.4% (89 out of 103 patients). With the limits of data interpretation due to the differences in the lengths of follow-up ranging from 1 to 21 months, seizure freedom was achieved by 45% (50 out of 111) of all patients.

Interestingly, recent studies have shed light on the potential role of tumor molecular markers, such as IDH1 mutation and MGMT methylation in seizure occurrence in patients with gliomas [41]. More specifically, the mutated form of IDH1 generates 2-hydroxyglutarate (2-HG), which is an oncometabolite and structurally resembles glutamate; it has been hypothesized that 2-HG may promote seizures by functioning as a glutamate receptor agonist [42]. However, although promising, clinical evidence on this topic is contrasting, with three studies [26,28,30] suggesting better seizure control with PER in BTRE patients with IDH1 mutation and MGMT methylation, and two studies pointing out no significant difference between the two molecular signatures. Additionally, the PER antitumor effect, hypothesized in several in vitro studies, has been studied in only one clinical study [25]. Future clinical trials and observational studies involving larger cohorts of patients are strongly warranted to better explore these interesting and promising relationships.

Add-on therapy with PER in patients with BTRE was generally well tolerated, and the rate of treatment discontinuation due to adverse events across the studies was 6.3% (eight out of 128 patients). Regarding the titration scheme, the overall low incidence of adverse effects reported with a 2-week up-titration suggests that a slow titration may represent a good strategy to minimize the risk of side effects. Shortcomings in the interpretation of findings related to tolerability included the variable and sometimes short lengths of the follow-up, and the ascertainment of adverse events occurrence through patients’ subjective reports in almost all studies; the study by Maschio et al. [29] was the only one to propose a more accurate self-report multi-item questionnaire to collect adverse events. Importantly, the PERADET study [30]—the only multicentric and prospective study available so far—evaluated the quality of life of patients with BTRE before and during the treatment with PER, and showed no changes; these findings further supported the overall good tolerability of PER. Additional issues need to be acknowledged for evaluating the tolerability of ASMs in BTRE. First, the adverse effects of ASMs are known to be more frequent in patients with BTRE, and the adverse effects of anticancer therapies could heavily influence their tolerability, exacerbating or even triggering their unwanted effects (e.g., nausea) [2]. Moreover, tumor progression may act as an important confounding factor, potentially being the physical and/or psychological contributor to some of the adverse effects attributed to ASMs (e.g., headache, dizziness, anxiety). Reasonably, an ASM can be considered responsible for the adverse effects when they occur at the initiation of the therapy, after an increase in drug dosage, or when they disappear after discontinuing treatment.

Selecting the appropriate ASM in patients with BTRE is challenging and several issues need to be considered, such as the risk of drug-drug interactions, concurrent antitumor treatments, the cognitive status of patients and other neurological symptoms. Levetiracetam has been suggested to be the preferred choice as first-line treatment in patients with BTRE [43,44]; in case of poor seizure control with monotherapy, potential add-on ASMs include lacosamide, perampanel, and valproic acid [44]. First-generation ASMs (e.g., carbamazepine, phenobarbital, phenytoin and valproic acid) are well known to cause drug-drug interactions: chemotherapeutic agents can reduce the concentration of first-generation ASMs and these ASMs can reduce the activity of chemotherapeutic agents. Moreover, phenytoin and phenobarbital shorten the half-life and increase the total body clearance of dexamethasone and prednisone [45]. Valproic acid, an enzyme inhibitor, can increase the toxicity of cisplatin, etoposide and nitrosoureas, and it has been also associated with coagulopathy, especially thrombocytopenia, which may worsen in combination with chemotherapy [46]. Because of extensive hepatic metabolism, PER could be prone to interactions with cytochrome P450 substrates. In fact, it is known that the concomitant use of known moderate or strong CYP3A4 inducers, such as carbamazepine, phenytoin, and oxcarbazepine, can decrease the plasma levels of PER by approximately 50% to 67% [47]. In contrast, PER itself does not seem to show significant enzyme inhibition or induction [48]. So far, drug interactions leading to a worsening of the tolerability of PER in BTRE have not been reported.

## 5. Conclusions

Currently available evidence, despite being based on a few non-randomized, non-controlled studies characterized by small sample sizes and open to high risk of bias, suggests the role of PER as a viable and effective therapeutic option to improve seizure control in patients with BTRE. Although present and not negligible, adverse effects are generally of modest impact and do not greatly impact treatment retention. In vitro studies indicated promising antitumor properties of PER, while clinical data on its potential antitumor activity are still scarce. Additional controlled trials and observational studies in larger cohorts are needed to further explore the effects of PER on tumor progression and fully characterize its potentialities in patients with BTRE. To date, one phase I-II, interventional, open-label, pilot trial is ongoing to measure the effect of PER on peritumoral hyperexcitability using intraoperative electrocorticography at the time of initial glioma resection as well as seizure control in patients with newly diagnosed high-grade gliomas compared to standard of care treatment [49]. A phase IV clinical trial in adult patients with biopsy-proven high-grade glioma and focal epilepsy is also ongoing to evaluate the efficacy and safety of PER compared with alternate ASMs, assess the change in neurocognitive function and brain magnetic resonance imaging progression over the course of PER treatment, and identify a biomarker-specific response to seizure-reduction [50].

## Figures and Tables

**Table 1:** Main characteristics and findings of pre-clinical studies.

Study (Year)	Study Design	Main Findings
Cunningham (2016)	Ex vivo	PER blocked inter-ictal discharges in ex vivo human peritumoral brain slices
Lange et al. (2019)	In vitro	PER showed systematic inhibitory effects on cell proliferation in patient-derived low-passage cell lines of glioblastoma. Metastasis cells were more resistant to PER. Glucose uptake was attenuated in all glioblastoma cells after exposure to PER, whereas apoptosis was not induced
Lai et al. (2019)	In vitro	PER suppressed voltage-gated Na+ currents in U87 glioma cells
Mayer et al. (2019)	In vitro, in vivo	PER reduced glucose uptake in vitro without affecting extracellular glutamate levels. PER prevented recurrent epileptiform discharges in brain slices from rats bearing C6 glioma. PER did not reduce tumor size
Lange et al. (2020)	In vivo	PER showed anticonvulsant properties in rodent F98 glioma model. PER as an add-on treatment to radiochemotherapy had no effect on tumor progression, but preserved the glutamatergic network activity on healthy peritumoral tissue
Salmaggi et al. (2021)	In vitro	PER showed antitumor activity in glioblastoma cell lines U87, U138, A172 and the grade III astrocytoma cell line SW1783, via a pro-apoptotic effect. The combination of PER and temozolomide had a significant synergistic effect
Tatsuoka et al. (2022)	In vitro	PER showed inhibitory effects on cell viability in a dose-dependent manner on malignant glioma cell lines A-172, AM-38, T98G, U-138MG, U-251MG and YH-13, via a pro-apoptotic effect
Yagi et al. (2022)	In vitro	Inhibitory effect on cell proliferation of PER confirmed on A-172, AM-38, T98G, U-138MG, U-251MG and YH-13 cell lines. PER and temozolomide showed synergistic effect on T98G and U-251MG lines. PER suppressed migration of T98G and U-251MG cells.

Abbreviation: PER = perampanel.

**Table 2:** Main characteristics of clinical studies and efficacy findings of perampanel in patients with brain tumor-related epilepsy.

Study (Year)	Study Design	Number of Participants	Main Efficacy Findings
Vecht et al. (2017)	Prospective study	12	Objective seizure response in 9 out of 12 patients (75%): 50% seizure reduction in 3 patients and seizure-freedom in 6 patients
Izumoto et al. (2018)	Case series	12	Seizure frequency reduction ≥50% in 12/12 patients, with six patients (60%) seizure free
Dunn-Pirio et al. (2018)	Prospective study	8	Self-reported seizure reduction in 6 out of 8 patients; percent seizure reduction not available as baseline seizure frequencies were not collected. No participants reached seizure freedom
Maschio et al. (2019)	Retrospective study	11	After 12 months of PER add-on therapy, 5 patients were seizure-free, 4 had ≥50% seizure frequency reduction, and seizure frequency was unchanged in 2 patients. The responder rate was 81.8%. The final median dose of PER was 7.3 mg/day
Chonan et al. (2020)	Retrospective study	18	All patients were receiving LEV monotherapy. Seventeen of 18 patients achieved seizure freedom with 2–4 mg of PER. The median time to achieve seizure freedom after PER add-on was 11 days (range 0–2 months)
Maschio et al. (2020)	Prospective study	26	After 6 months of follow-up, 8 patients were seizure-free, 15 had ≥50% seizure reduction, and 3 remained stable. Five patients dropped out. No significant differences was found in seizure control in patients with/without IDH1 mutation and MGMT methylation
Coppola et al. (2020)	Prospective study	36	After 12 months of follow-up, 21 patients were available for evaluation, with a responder rate of 90.4% and 33.3% of patients being seizure-free
Heugenhauser et al. (2021)	Case series	5	The responder rate was 40%, with 2 patients experiencing a ≥50% reduction in seizure frequency after add-on treatment with PER. One patient was seizure-free after 1 year

Abbreviation: PER = perampanel.

**Table 3:** Main tolerability findings of perampanel in patients with brain tumor-related epilepsy.

Study (Year)	Patients with Adverse Effects (%)	Adverse Effects (% of Patients)	Discontinuation of PER Due to Adverse Effects (% of Patients)
Vecht et al. (2017)	6 (50)	Dizziness (33), drowsiness (17)	1 (8)
Izumoto et al. (2018)	2 (17)	Dizziness (17)	1 (8)
Dunn-Pirio et al. (2018)	NR	Fatigue (63), dizziness (25), confusion (13), nausea (13), somnolence (13), insomnia (NR), anxiety (NR)	1 (13)
Maschio et al. (2019)	2 (18)	Anxiety (9), agitation (9)	0 (0)
Chonan et al. (2020)	2 (11)	Irritability (11)	0 (0)
Maschio et al. (2020)	4 (15)	Vertigo (7.5), aggressiveness (7.5)	2 (8)
Coppola et al. (2020)	11 (31)	Anxiety (6), aggressiveness (6), dizziness (14), fatigue (6)	3 (27)
Heugenhauser et al. (2021)	2 (40)	Fatigue (20), aggressiveness (20)	0 (0)

Abbreviations: NR = not reported; PER = perampanel.

## Data Availability

Data sharing is not applicable to this article as no datasets were generated or analyzed during the current study.
